# Colostrum insulin supplementation does not influence immunoglobulin G absorption in neonatal Holstein bulls

**DOI:** 10.3168/jdsc.2022-0351

**Published:** 2023-03-16

**Authors:** K.S. Hare, K.M. Wood, R. Sargent, M.A. Steele

**Affiliations:** 1 Department of Animal Biosciences, Animal Science and Nutrition, Ontario Agricultural College, University of Guelph, Guelph, ON, Canada N1G 1Y2; 2 Saskatoon Colostrum Company Ltd., Saskatoon, SK, Canada S7K 6A2

## Abstract

Since insulin has been demonstrated to suppress IgG absorption in other neonatal species, we had the objective to delineate how colostral insulin concentrations affect IgG absorption in neonatal bovines. We enrolled Holstein bull calves (n = 48; body weight = 46.3 ± 0.84 kg) at birth and randomized them by birth order to receive (1) colostrum that contained basal insulin concentrations (12.9 μg/L; n = 16), or colostrum that had been supplemented with an exogenous insulin to increase the insulin concentration to either (2) 5 times (70.0 μg/L; n = 16) or (3) 10 times (149.7 μg/L; n = 16) that of the basal colostrum. Gross colostrum composition (crude fat: 4.1 ± 0.06%; crude protein: 11.7 ± 0.05%; lactose: 1.9 ± 0.01%; IgG: 63.9 ± 1.19 g/L) was similar between treatments and calves were fed (7% body weight, 3.1 ± 0.06 L) their treatments at 2, 14, and 26 h postnatal. Serum was collected at 0, 30, 60, 90, 120, 180, 240, 360, 480, and 600 min postprandial respective to the first and second colostrum feeding and analyzed for IgG concentration. The incremental area under the curve (I-AUC) and apparent efficiency of absorption (AEA) were calculated for the 10-h periods following the first and second colostrum meal. Serum IgG concentrations over time, I-AUC, and AEA were statistically analyzed as a complete randomized design. Colostrum insulin concentration did not affect serum IgG concentrations or the I-AUC or AEA after calves were fed colostrum at 2 and 14 h postnatal. High colostral insulin content is not detrimental or promotive to IgG absorption in neonatal Holstein bulls.

## Highlights

• Colostrum contains elevated insulin concentrations that are highly variable.
• Immunoglobulin G absorption in neonatal calves is not affected by insulin feeding.
• High colostrum insulin concentrations are not a risk factor for poor IgG absorption.

## Graphical Abstract

**Figure:**
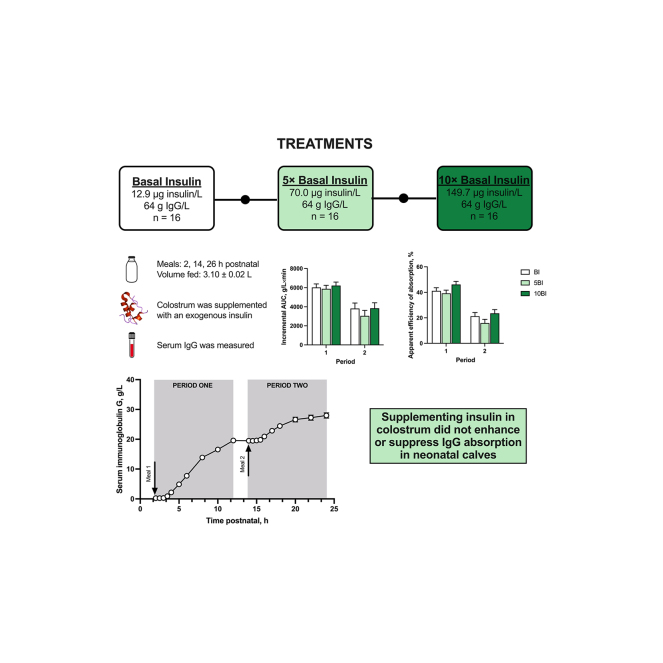
**Summary:** We enrolled 48 neonatal Holstein bulls to evaluate how linear increases in colostrum insulin concentration (from 12.9 to 149.7 μg of insulin/L) would influence immunoglobulin G (IgG) absorption in neonatal calves. At 2, 14, and 26 hours postnatal, calves were fed nonsupplemented colostrum or colostrum that had been supplemented with insulin and we analyzed serum IgG concentration at 0, 30, 60, 90, 120, 180, 240, 360, 480, and 600 minutes relative to the first and second meal. Colostral insulin concentration did not affect IgG absorption.

Neonatal calves initially derive passive immunity from ingestion and absorption of colostral IgG. Sufficient IgG absorption is required to reduce the incidence of early life morbidity and mortality (Lombard et al., 2020). The sufficiency of IgG absorption has recently been classified as poor (<10.0 g of IgG/L), fair (10.0 to 17.9 g of IgG/L), good (18.0 to 24.9 g of IgG/L), and excellent (≥25.0 g of IgG/L; Lombard et al., 2020) respective to serum IgG concentrations. Predominantly, IgG concentration and mass of IgG consumed (as a function of IgG concentration and volume fed) determine serum IgG concentration (Godden et al., 2009; Lopez et al., 2022). Yet, numerous factors affect the absorption of colostral IgG relating to colostrum management (Elizondo-Salazar and Heinrichs, 2009; Fischer et al., 2018; Hare et al., 2020) and composition (Balfour and Comline, 1962; Cabral et al., 2014). It is possible that bioactive components present in colostrum may affect colostral IgG absorption (Schlagheck, 1983), as colostrum contains numerous bioactive compounds (reviewed in Fischer-Tlustos et al., 2021), such as growth factors and peptide hormones, oligosaccharides, biologically active fatty acids, and microRNA, that have not been investigated for their relationship to IgG absorption in calves. In other neonatal species, other humoral and colostral compounds have been shown to alter the absorption of colostral IgG (for instance, epidermal growth factor reduced IgG absorption in neonatal rat pups; Harada et al., 1990, 1994a,b).

Colostrum contains elevated insulin concentrations (∼55 to 100 μg/L; Zinicola and Bicalho, 2019) relative to blood (Mann et al., 2016) that exhibit high intersubject variation (6 to 327 μg/L; Malven et al., 1987; Aranda et al., 1991). Colostrum insulin is of interest, as insulin has been found to influence IgG absorption in adrenalectomized neonatal rodents (Harada and Syuto, 1991). In neonatal piglets, insulin administration shortly after birth was also shown to suppress the transfer of BSA, a fluorescein isothiocyanate-labeled dextran, and sow colostral IgG (Svendsen et al., 1986). The authors speculated that insulin regulates intestinal closure and impermeability to IgG by inducing the synthesis of structural proteins in the enterocyte membrane (Svendsen et al., 1986). Tyler and Ramsey (1993) also evaluated how insulin affected macromolecular transfer in neonatal calves and contrastingly observed that insulin injection did not affect peak IgG concentrations, but delayed the time of intestinal closure, speculating that this response was due to a hypoglycemic state. Collectively, it is unclear if colostrum-derived insulin will promote or blunt the absorption of IgG in neonatal calves. However, it is possible that colostrum-derived insulin may inhibit IgG absorption as it is likely to act luminally without affecting peripheral glucose concentrations (Hare et al., 2023). Since bovine colostrum contains elevated but highly variable insulin concentrations (6 to 327 μg of insulin/L; Malven et al., 1987; Aranda et al., 1991), it is important to consider how colostrum-derived exogenous insulin may affect the absorption of colostral IgG in neonatal calves.

We hypothesized that orally consumed exogenous insulin supplemented in colostrum would decrease the absorption of IgG in neonatal Holstein bulls. Our objective was to evaluate how supplementing insulin in colostrum to 5 and 10 times the basal colostral insulin concentration would affect IgG concentrations over time, the IgG area under the curve (**AUC**), and apparent efficiency of absorption (**AEA**) after colostrum feeding.

The experiment was conducted in accordance with Canadian Council of Animal Care guidelines and approved by the University of Guelph (Guelph, ON) Animal Care Council (Animal Utilization Protocol #4126). We previously described the experimental protocol and animal enrollment in Hare et al. (2023). In brief, neonatal Holstein bulls (n = 48; BW: 46.3 ± 0.8 kg) from multiparous Holstein-Friesian cows were sourced from a commercial farm in Southwestern Ontario. The cow and calf were carefully monitored after parturition to ensure that calves did not suckle and were separated by 60 min postnatal. Calves were then weighed using a digital platform floor scale (PS2000; Brecknell-Scale, Avery-Weigh Tronix), towel-dried, and housed in calf pens (1.22 m^2^) bedded deeply with wheat straw.

The study was conducted as a completely randomized design and research personnel randomized and stratified treatments by birth order in advance of the study (complete randomized design). They were fed a pooled and heat-treated colostrum at 2, 14, and 26 h postnatal that contained: (1) basal colostrum insulin concentrations (**BI**; 12.9 μg of insulin/L; n = 16); (2) a 5-fold (**5BI**; 60.0 μg of insulin/L; n = 16) increase in colostrum insulin respective to BI; or (3) a 10-fold (**10BI**; 149.7 μg of insulin/L; n = 16) increase in colostrum insulin concentration respective to BI. Blinding was not possible because insulin was supplemented in colostrum at the time of feeding (described below). Replication was determined as described in Hare et al. (2023). The basal colostrum was formulated with Saskatoon Colostrum Company Ltd. (Saskatoon, SK, Canada) by pooling and heat-treating 112 colostrum sources that contained >40 g of IgG/L and low insulin concentrations (median: 18.6 μg of insulin/L). Once pooled and heat-treated, the basal colostrum macronutrient and insulin content were re-analyzed and are reported in Table 1. Insulin was supplemented in the 5BI and 10BI treatments using a 1:5 ratio of fluorescein isothiocyanate-labeled human insulin (Sigma-I3611; Millipore-Sigma) and unlabeled bovine insulin (Sigma-I0516, Millipore-Sigma) and 5BI and 10BI insulin doses were chosen to be within range of the colostral insulin distribution that has been observed (Malven et al., 1987; Blum and Hammon, 2000; Zinicola and Bicalho, 2019). The fluorescein isothiocyanate-labeled human insulin was added to address objectives detailed in Hare et al. (2023). Calves were fed (7% BW; 3.10 ± 0.02 L) their respective colostrum treatments at 2 h 14 min, 14 h 5 min, and 26 h 6 min postnatal using an esophageal tube (Godden et al., 2009; Desjardins-Morrissette et al., 2018). The meal at 26 h postnatal was provided to ensure that no retrogressive gastrointestinal atrophy occurred before an intestinal dissection performed at 30 h (Hare et al., 2023). A 40-mL colostrum sample was collected at each meal and frozen at −20°C until analysis (described below).

**Table 1:** Feeding rate, colostrum intake and composition, and characteristics of calves that consumed colostrum at 2, 14, and 26 h postnatal that had either basal insulin (BI; 12.9 μg/L) concentration or were supplemented with an exogenous insulin to 5× (5BI; 70.0 μg/L) or 10× (10BI; 149.7 μg/L) the BI concentration^1^

Parameter	Treatment			SE	*P*-value	
	BI	5BI	10BI		Linear	Quadratic
Feeding rate, %/kg of BW	7.0	7.0	7.0	—	—	—
Meal mass, kg	3.2	3.3	3.2	0.06	0.78	0.58
Meal volume, L	3.1	3.1	3.1	0.06	0.66	0.59
Specific gravity, kg/L	1.04	1.04	1.04	0.002	0.36	0.52
Colostrum composition						
Crude fat, %	4.1	4.1	4.1	0.06	0.74	0.65
CP, %	11.7	11.7	11.6	0.05	0.74	0.61
Lactose, %	1.9	1.9	1.9	0.01	0.54	0.76
TS, %	19.6	19.6	19.6	0.08	0.81	0.81
Urea, mg/dL	35.4	35.1	35.2	0.35	0.64	0.69
BHB, m*M*	0.23	0.23	0.24	0.010	0.45	0.73
SCC, ×1,000 cells/mL	148.2	138.0	136.1	7.30	0.21	0.70
IgG, g/L	63.9	63.5	64.2	1.19	0.86	0.76
Insulin,^2^, ^3^ μg/L	12.87	70.0	149.65	2.104	<0.001	<0.001
IgG intake, g/meal	196.9	197.5	198.1	5.30	0.87	1.00
Calf characteristic						
Start BW, kg	45.9	46.6	46.3	0.84	0.70	0.64
Frame measurement, cm						
Body length	63.7	64.6	63.9	0.62	0.75	0.31
Chest circumference	84.2	85.2	84.5	0.58	0.65	0.24
Withers height	81.2	83.1	82.6	0.70	0.12	0.18
Hip height	84.0	86.4	86.1	0.84	0.054	0.24

1 Reproduced from Hare et al. (2023).

2 The endogenous colostrum insulin content for the 5BI and 10BI treatments is equivalent to the BI treatment.

3 Colostrum insulin content was increased in the 5BI and 10BI treatments using 4 parts exogenous unlabeled bovine insulin (Sigma-I0516; Millipore-Sigma) and 1 part fluorescein isothiocyanate-labeled human insulin (Sigma-I3611; Millipore-Sigma) respective to an anticipated endogenous colostrum insulin content of 16.8 μg/L.

Using a venous jugular catheter, blood was collected from calves at 0, 30, 60, 90, 120, 180, 240, 360, 480, and 600 min relative to the first and second colostrum feeding. The volume of blood collected at each time point was proportional to calf BW (average: 15.8 ± 0.2 mL; range: 14 to 18 mL) and standardized so that the total volume of blood collected over the 24-h sampling period did not exceed 1% calf birth BW (vol/wt). We standardized collection volume respective to calf BW to (1) collect the maximal volume allowed by our animal utilization protocol to ensure that there was adequate supernatant for all laboratory analysis, and (2) the calculation for AEA of IgG uses a constant plasma volume (9.1% BW; Quigley et al., 1998), necessitating standardizing blood collection volume by BW to increase precision in calculating AEA between calves differing in BW. Serum was separated by centrifugation (920 × *g* for 25 min at 4°C) in a Vacutainer (BD366480; Becton Dickinson) after allowing blood to clot for 30 min at room temperature. Serum supernatant was aliquoted (1.5 mL/aliquot) to 3 microcentrifuge tubes and stored at −20°C until analysis.

Colostrum was analyzed for gross composition (crude fat, CP, lactose, TS, urea, BHB, and SCC) by mid-infrared spectroscopy (Lactanet, DHI) using a 1:3 colostrum:double-distilled H_2_O dilution ratio. Colostral and serum IgG concentrations were determined by the Saskatoon Colostrum Company Quality Assurance Laboratory (Saskatoon Colostrum Company Ltd.) using radial immunodiffusion analysis (Shivley et al., 2018).

Incremental AUC (**I-AUC**; Chiou, 1978; Cardoso et al., 2011) and IgG AEA (Quigley et al., 1998) from 2 to 12 h and 14 to 24 h postnatal were calculated for serum IgG during the 10-h measurement periods after the first (2 h postnatal) and second (14 h postnatal) colostrum feedings. Calves were categorized as having poor (<10 g of IgG/L), fair (10 to 17.9 g of IgG/L), good (18 to 24.9 g of IgG/L), or excellent (≥25 g of IgG/L; Lombard et al., 2020) transfer of passive immunity using serum IgG concentrations at 24 h.

Calves in this study experienced unintended variations in environmental ambient temperature (described in Hare et al., 2023) and a discrete blocking factor was introduced post hoc to account for model variations due to ambient temperature. Calves were stratified into 5 discrete blocks (−11.0 to −3°C, n = 4; −3.0 to 5.0°C, n = 15; 5.0 to 13.0°C, n = 5; 13.0 to 21.0°C, n = 12; and 21 to 29°C, n = 12) based on the average ambient temperature that they were exposed to (from hourly data over the 30 h from birth to experiment completion). Each treatment was represented within each block.

All data were assessed for residual distribution to determine whether their residuals were normally, independently, and identically distributed. Normality was assessed with PROC UNIVARIATE (SAS 9.4; SAS Institute Inc.) and confirmed normally distributed by Shapiro-Wilk *P* > 0.05. Non-Gaussian distributions (Shapiro-Wilk *P* < 0.05) were corrected using a lognormal distribution specified in the MODEL statement of PROC GLIMMIX. Homogeneous residual variance between treatments was tested using the COVTEST statement in PROC GLIMMIX and residuals were confirmed homoscedastic when the homogeneity *P* > χ^2^ was >0.05. Heteroscedasticity was accounted for using the GROUP = command in the RANDOM statement of PROC GLIMMIX to specify that residual variance be grouped by treatment within the model. Data were then analyzed as a complete randomized design with PROC GLIMMIX including the fixed effects of treatment (BI, 5BI, and 10BI) and block and random effect of calf (treatment) specified to the R-sided random effects matrix. Unique contrast coefficients based on post hoc insulin supplementation generated using PROC IMP were used to estimate the linear and quadratic treatment effects. For IgG concentration over time, the fixed effects of time and the treatment by time interaction were added to the model. Autocorrelation was accounted for by modeling covariance and selecting the best-fit covariance structure (by the lowest Akaike information criterion and Bayesian information criterion values). Degrees of freedom were approximated using Kenward-Rogers, and Tukey-Kramer and Games-Howell post hoc adjustments were used for multiple comparison tests with nonrepeated and unevenly spaced repeated measurements, respectively. Categorical data (classification of serum IgG concentrations at 24 h) were modeled using PROC FREQ. Differences are declared when *P* < 0.05 and tendencies are considered when 0.05 ≤ *P* < 0.10.

As discussed in Hare et al. (2023), colostrum intake (mass and volume) and gross composition were similar (*P* ≥ 0.21; Table 1) between treatments. Colostral IgG concentration (63.9 to 64.2 ± 1.19 g of IgG/L) did not differ (*P* = 0.86) between treatments, nor did IgG intake (*P* = 87). Only colostral insulin concentration differed (*P* < 0.001) between BI (12.9 μg of insulin/L), 5BI (70.0 μg of insulin/L), and 10BI (149.7 μg of insulin/L) treatments.

Baseline serum IgG concentrations were not different (*P* = 0.27; Table 2) between treatments before the first colostrum meal and remained similar (*P* = 0.57) before the second. Following either meal, maximal serum IgG concentrations (C_max_) did not differ (*P* ≥ 0.30) by insulin treatment. The I-AUC did not differ (*P* ≥ 0.49) for BI, 5BI, and 10BI calves after the 2- and 14-h colostrum feedings, nor (linear: *P* = 0.66) did the IgG AEA. A tendency for a quadratic effect (*P* = 0.055) of treatment on IgG AEA during the second postprandial period was detected. Although serum IgG concentrations did not differ by treatment over time (treatment × time: *P* ≥ 0.50; Figure 1), serum IgG concentrations consistently increased (time: *P* < 0.001) after the first and second colostrum feeding. No differences (*P* = 0.44) were observed in serum IgG categories. “Excellent” scores proportionally ranged from 62.5 (BI) to 81.3% (10BI), whereas “good” scores proportionally ranged from 37.5 (BI) to 18.8% (5BI and 10BI); 6.3% of 5BI calves had “fair” scores.

**Table 2:** Serum IgG parameters of calves that consumed colostrum at 2, 14, and 26 h postnatal that had either basal insulin (BI; 12.9 μg/L) concentration or were supplemented with an exogenous insulin to 5× (5BI; 70.0 μg/L) or 10× (10BI; 149.7 μg/L) the BI concentration

Parameter	Treatment			SEM	*P*-value	
	BI	5BI	10BI		Linear	Quadratic
Period 1^1^						
Baseline, g/L	0.26	0.29	0.25	0.093	0.25	0.87
C_max_, g IgG/L	20.4	20.1	22.0	1.07	0.30	0.38
I-AUC, g/L × min	6,032.7	5,884.8	6,226.4	364.59	0.71	0.59
AEA, %	41.2	39.3	46.2	2.49	0.15	0.16
Period 2^1^						
Baseline, g/L	20.0	19.8	19.1	1.14	0.57	0.88
C_max_, g IgG/L	32.2	31.7	32.2	1.51	0.83	0.71
I-AUC, g/L × min	3,822.0	3,049.8	3,849.0	574.93	0.49	0.8
AEA, %	21.3	15.9	23.6	2.93	0.66	0.055
Transfer of passive immunity category^2^, ^3^, ^4^						
Excellent	62.5	75.0	81.3	—	—	—
Good	37.5	18.8	18.8	—	—	—
Fair	0.0	6.3	0.0	—	—	—

1 Period 1 is the 10 h postprandial of the first colostrum feeding at 2 h postnatal, and period 2 is the 10 h postprandial of the second colostrum feeding at 14 h postnatal. C_max_ = maximal serum IgG concentration; I-AUC = incremental area under the curve; AEA = apparent efficiency of absorption.

2 Calves were categorized at 24 h postnatal accordingly: excellent, serum IgG ≥25 g/L; good, serum IgG >18 g/L and <24.9 g/L; fair, serum IgG >10 g/L and <17.9 g/L; and poor, serum IgG <10 g/L (Lombard et al., 2020).

3 No calves within any treatment were classified as having poor serum IgG concentrations at 24 h postnatal.

4 χ^2^*P*-value = 0.44.

**Figure 1:**
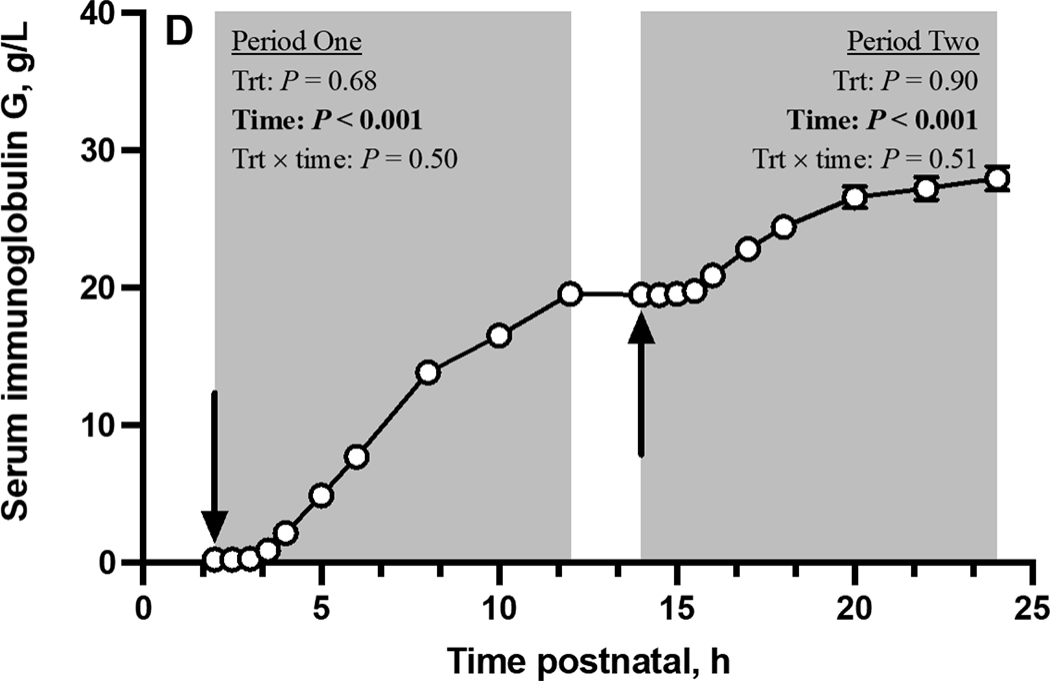
Serum IgG concentration (SE: 0.49 g/L) over time of neonatal Holstein bulls that consumed colostrum at 2, 14, and 26 h postnatal that had either basal insulin (BI) concentration (12.9 μg of insulin/L; n = 16) or were supplemented with an exogenous insulin to 5× (70.0 μg of insulin/L; n = 16) or 10× (149.7 μg of insulin/L; n = 16) the BI concentration. Period 1 is the postprandial period following the first meal at 2 h postnatal, whereas period 2 is the postprandial period following the second meal at 14 h postnatal. Black arrows denote when meals were fed at 2 and 14 h postnatal. Serum IgG differed (*P* < 0.001) with respect to time during the first and second postprandial period but was not affected (*P* ≥ 0.50) by treatment or the treatment by time interaction. Data are presented as LSM ± SE, and some error bars are too small to be seen.

The conditions that we applied in this experiment met our objectives of evaluating how colostral insulin concentration affected absorption of colostral IgG. We provided a pooled source of colostrum, ensuring that all calves did not differ in their macronutrient and IgG provision at 2, 14, or 26 h postnatal. Thus, IgG absorption by neonatal calves in this study was not confounded by differences in colostrum composition (Balfour and Comline, 1962), osmolality or pH (Cabral et al., 2014), or IgG intake (Hare et al., 2020). Contrary to our hypothesis, supplementing exogenous insulin in bovine colostrum to 5 and 10 times the basal concentration did not affect the absorption of IgG in neonatal Holstein bulls. No differences were observed between BI, 5BI, or 10BI calves with respect to serum IgG concentrations over time, IgG C_max_, IgG I-AUC, or IgG AEA following the first or second colostrum feeling, indicating that insulin feeding did not affect IgG absorption. This is further supported by calves not differing in their sufficiency of transfer of passive immunity.

A key difference between our study and others (Svendsen et al., 1986; Harada and Syuoto, 1991) is the animal model used. Differences in gastrointestinal physiology and macromolecular absorption between suckling rat pups, neonatal pigs, and neonatal calves could explain why insulin provision to neonatal calves did not influence IgG absorption. Whether administered parenterally (Svendsen et al., 1986; Tyler and Ramsey, 1993) or enterally (Harada and Syuoto, 1991), precocious cessation of IgG transport was accompanied by peripheral hyperinsulinemia with or without concurrent hypoglycemia (Harada and Syuoto, 1991; Tyler and Ramsey, 1993). The neonatal intestine in suckling rodents (Mosinger et al., 1959; Kelly, 1960) and piglets (Asplund et al., 1962; Shen and Xu, 2000) is permeable to enteral insulin, causing hyperinsulinemia and hypoglycemia. However, in neonatal calves, the intestine does not appear to be permeable to insulin (Hare et al., 2023) unless insulin is provided orally in advance of nutritive feeding (Kirovski et al., 2008) or when pharmacological insulin doses are combined with milk (Pierce et al., 1964). Hare et al. (2023) did not observe that linear increases in colostrum insulin concentration increased plasma insulin concentration to induce concurrent decreases in plasma glucose concentrations; although, plasma glucose appeared more quickly, and serum nonesterified fatty acids were cleared more quickly with increased colostrum insulin concentration. Speculatively, induction of peripheral hyperinsulinemia independent of hypoglycemia, whether by insulin injection or gastrointestinal absorption, may be required to prompt premature intestinal closure. In other words, ad luminal rather than luminal hyperinsulinemia might decrease IgG absorption. The dose of colostrum insulin in this study mimicked the natural range found in colostrum (Malven et al., 1987; Blum and Hammon, 2000; Zinicola and Bicalho, 2019) and did not influence peripheral insulin or glucose concentrations in neonatal calves, but rather acted locally within the gastrointestinal tract (Hare et al., 2023), thereby explaining why colostral insulin supplementation did not affect IgG absorption in neonatal calves.

After feeding supplemental insulin in colostrum for a sustained period, we concluded that colostral insulin does not affect IgG absorption in neonatal Holstein bulls. Elevated colostral insulin concentrations are not a risk factor for poor transfer of passive immunity.
